# Importance of maintaining redox potential balance in the development of type 2 diabetes

**DOI:** 10.1186/1753-6561-6-S3-P39

**Published:** 2012-06-01

**Authors:** Hava Rozenfeld, Moria Chetboun, Sanford R Sampson, Tovit Rosenzweig

**Affiliations:** 1 Department of Molecular Biology, Department of Nutritional Studies, Ariel University Center, Israel; 2 Faculty of Life Sciences, Bar-llan University, Ramat-Gan, lsrael; 3 Department of Molecular Cell Biology, Weizmann Institute of Science, Rehovot, Israel

## Background

Accumulation of ROS leads to oxidative stress, which is a common denominator in many diseases. As a result of promising results obtained from *in-vitro* and *in-vivo* studies showing the beneficial function of antioxidants in the prevention of diseases, including benefits of antioxidants on insulin sensitivity and β-cell viability and function, supplementation of antioxidants became very popular. Unfortunately, major randomized clinical trials have yielded disappointing results. Meta analyses conclude that antioxidant supplementations have no beneficial effects on the prevalence of type 2 diabetes (T2D). There are several possible explanations for the failure of antioxidant supplementation to improve health outcomes. Our hypothesis is that there is an optimal redox state that should normally be maintained in cells. If shifted either to the oxidized or to the reduced state, disturbances in β-cell function and in insulin sensitivity of target tissues appear. The aim of this study is to clarify the correlation between redox potential and the development of diabetes.

## Materials and methods

Both *in-vitro* and *in-vivo* experiments were conducted. *In-vitro* study was performed on insulinoma RinM and Min6 cell lines. The effects of H_2_O_2_ and the antioxidant N-acetyl-L-cysteine (NAC) at different concentration were investigated on insulin secretion, viability of β-cells and mRNA expression of specific β-cell transcription factors. *In-vivo* experiments were conducted on KK-A^y^ mice, a known model of T2D. Mice were given NAC at different concentrations (200-1800 mg/kg/day) during adulthood. Diabetes was evaluated in treated mice by glucose and insulin tolerance tests, histological studies of pancreatic β-cells; insulin signaling pathway was followed in muscle tissue, and several analyses were determined in plasma (insulin, TBARS, TG, cholesterol).

## Results

*In-vitro* experiments show that, whereas high concentrations of H_2_O_2_ (>10 µM) induce oxidative stress and pancreatic β-cell death, low concentrations (4 µM) increased viability of these cells, and basal and glucose-induced insulin secretion. High concentrations of NAC reduced viability of cells [1]. mRNA expression of Pdx1 and Pax4 is regulated by the redox state of cells. *In-vivo* results show that while 600, 1200 and 1800 mg/kg/day NAC were all found to improve glucose tolerance of mice, the 1200 mg/kg/day treatment was the most effective in improving insulin sensitivity as indicated by low HOMA-IR. This study clarifies the dose-response effect of NAC on the development of diabetes, and on other manifestations of the metabolic syndrome.

## Conclusions

We conclude that alterations in redox balance, resulting from oxidative stress as well as oversupply of antioxidants, may lead to disturbances in the function of pancreatic β-cells and of insulin target tissues.

The study clarifies the beneficial effects of NAC on insulin sensitivity and β-cell function, and suggests that excessive antioxidant consumption has deleterious effects on the development of diabetes. This may provide an explanation for the failure of intervention studies to achieve beneficial health outcomes, and may lead to personalized-adjusted consumption of antioxidants.
